# Mechanisms Underlying Disorders of Consciousness: Bridging Gaps to Move Toward an Integrated Translational Science

**DOI:** 10.1007/s12028-021-01281-6

**Published:** 2021-07-08

**Authors:** Andrea I. Luppi, Joshua Cain, Lennart R. B. Spindler, Urszula J. Górska, Daniel Toker, Andrew E. Hudson, Emery N. Brown, Michael N. Diringer, Robert D. Stevens, Marcello Massimini, Martin M. Monti, Emmanuel A. Stamatakis, Melanie Boly, the Curing Coma Campaign and Its Contributing Collaborators

**Affiliations:** 1 Division of Anaesthesia, School of Clinical Medicine, University of Cambridge, Cambridge, UK; 2 Department of Clinical Neurosciences, University of Cambridge, Cambridge, UK; 3 Department of Psychology, University of California, Los Angeles, Los Angeles, CA USA; 4 Department of Psychiatry, University of Wisconsin–Madison, Madison, WI USA; 5 Department of Anesthesia and Perioperative Medicine, David Geffen School of Medicine, University of California, Los Angeles, Los Angeles, CA USA; 6 Department of Anesthesia, Critical Care, and Pain Medicine, Massachusetts General Hospital and Harvard Medical School, Harvard University, Boston, MA USA; 7 Department of Brain and Cognitive Sciences, Massachusetts Institute of Technology, Cambridge, MA USA; 8 Department of Neurology and Neurosurgery, Washington University in St. Louis, St. Louis, MO USA; 9 Departments of Anesthesiology and Critical Care Medicine, Neurology and Neurosurgery, and Radiology, School of Medicine, Johns Hopkins University, Baltimore, MD USA; 10 Dipartimento di Scienze Biomediche e Cliniche “L. Sacco”, Università Degli Studi Di Milano, Milan, Italy; 11 Istituto Di Ricovero e Cura a Carattere Scientifico, Fondazione Don Carlo Gnocchi, Milan, Italy; 12 Brain Injury Research Center, Department of Neurosurgery, David Geffen School of Medicine, University of California, Los Angeles, Los Angeles, CA USA

**Keywords:** Brain injury, Coma, Consciousness, Electroencephalography, Magnetic resonance imaging, Neuroimaging, Mechanism

## Abstract

**Aim:**

In order to successfully detect, classify, prognosticate, and develop targeted therapies for patients with disorders of consciousness (DOC), it is crucial to improve our mechanistic understanding of how severe brain injuries result in these disorders.

**Methods:**

To address this need, the Curing Coma Campaign convened a Mechanisms Sub-Group of the Coma Science Work Group (CSWG), aiming to identify the most pressing knowledge gaps and the most promising approaches to bridge them.

**Results:**

We identified a key conceptual gap in the need to differentiate the neural mechanisms of consciousness per se, from those underpinning connectedness to the environment and behavioral responsiveness. Further, we characterised three fundamental gaps in DOC research: (1) a lack of mechanistic integration between structural brain damage and abnormal brain function in DOC; (2) a lack of translational bridges between micro- and macro-scale neural phenomena; and (3) an incomplete exploration of possible synergies between data-driven and theory-driven approaches.

**Conclusion:**

In this white paper, we discuss research priorities that would enable us to begin to close these knowledge gaps. We propose that a fundamental step towards this goal will be to combine translational, multi-scale, and multimodal data, with new biomarkers, theory-driven approaches, and computational models, to produce an integrated account of neural mechanisms in DOC. Importantly, we envision that reciprocal interaction between domains will establish a “virtuous cycle,” leading towards a critical vantage point of integrated knowledge that will enable the advancement of the scientific understanding of DOC and consequently, an improvement of clinical practice.

## Introduction and Current State of Science

The last two decades have seen growing interest in the neuroscience of disorders of consciousness (DOC). Significant progress has led to the differentiation of clinical phenotypes, including the discovery of new syndromic entities such as cognitive-motor dissociation (CMD) syndrome, a condition characterized by behavioral unresponsiveness paired with evidence of covert consciousness (voluntary brain activity). At the same time, this progress has also highlighted the critical limitations in our practical ability to diagnose covert consciousness at the bedside, predict long-term trajectories and outcomes, and enhance neurological recovery with therapies that target specific biological mechanisms.

The discovery through functional magnetic resonance imaging (fMRI) studies [1–3] and scalp electroencephalography (EEG) [4] that ~15–20% of patients with DOC who lack overt behavioral responsiveness may nevertheless be covertly conscious highlights the critical need for diagnostic tools that rely on brain activity. Although some theory-driven approaches already seem promising [5, 6], further elucidation of the mechanisms underlying unconsciousness in DOC, as distinct from responsiveness, is a fundamental requirement to guide the development of new accurate bedside diagnostic tools. Additionally, almost 90% of patients with chronic DOC do not recover 1 year post injury [7]. Accordingly, there is a need to draw on large-scale and, ideally, longitudinal clinical studies to properly model prognostic trajectories. Although some new pharmacological (e.g., amantadine, zolpidem) and interventional therapies (e.g., deep brain stimulation [8], low-intensity-focused ultrasound [9]) have recently emerged for patients with DOC, both case reports and randomized controlled trials demonstrate only moderate success. Thus, a better mechanistic understanding of the different pathways leading to altered consciousness after brain injury could help develop more effective therapies as well as tailor more personalized treatment options to specific patients.

**Fig. 1:**
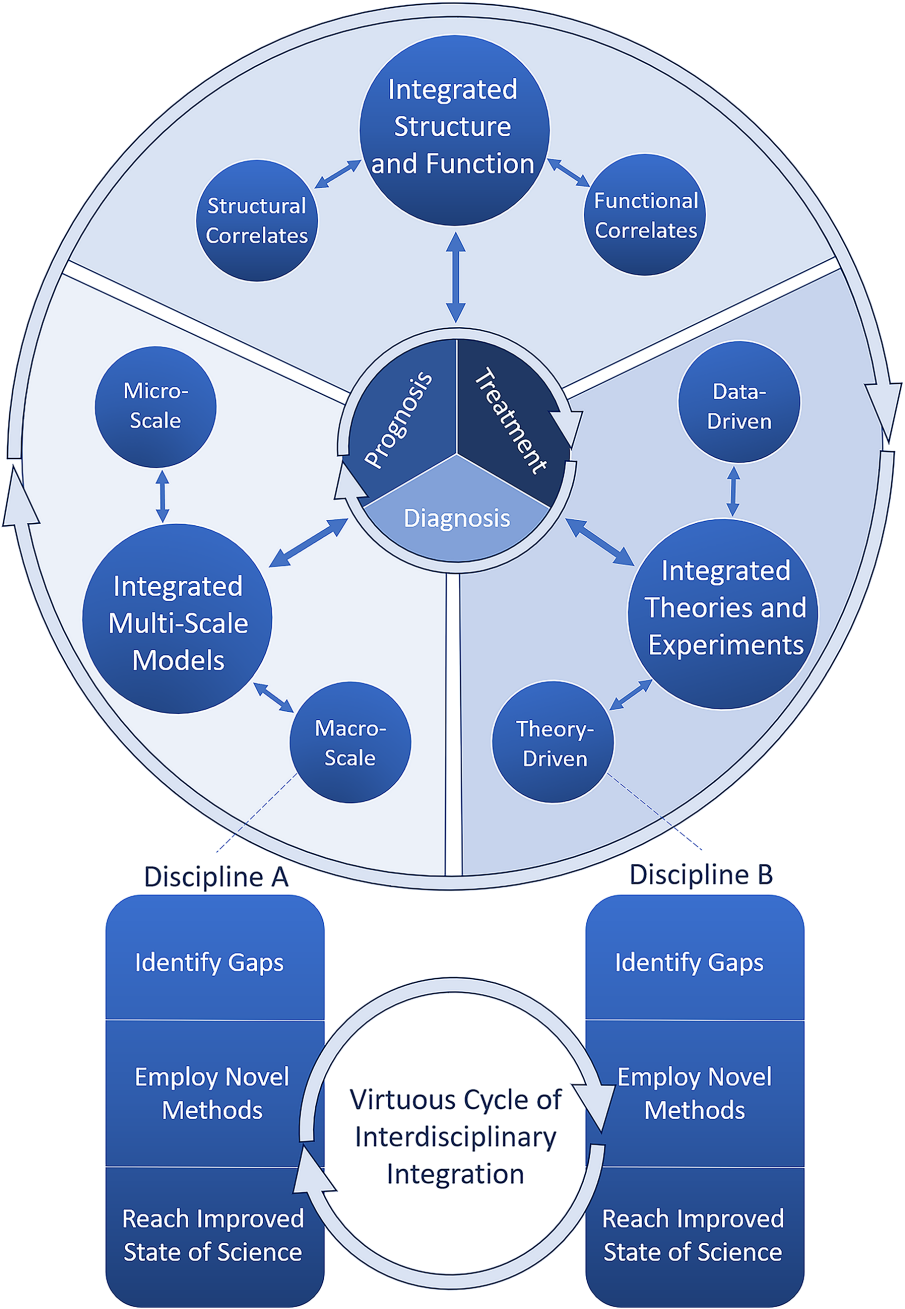
Overview of white paper recommendations. In this article, we have subdivided the gaps that exist in the field of disorders of consciousness (DOC) research into subdisciplines while stressing their mutual interdependence. The term “subdiscipline” is used for each branch of knowledge that makes up the study of DOC. Specifically, we suggest that efforts should be made to integrate structural and functional correlates, micro- and macroscale phenomena, and data- and theory-driven perspectives. Within each discipline (e.g., structural correlates), specific gaps should be identified and novel methods should be selected to answer these gaps and to reach an improved state of the science. Throughout this process, iterative integration with other disciplines is desired (bottom; note disciplines “A” and “B” can be replaced by any given subdiscipline of DOC research). Collectively, improved integration between these subfields of DOC is likely to provide the best avenue toward the clinical goals of DOC science: improved diagnosis, prognosis, and treatment (center circle). Circular arrows represent iterative processes, whereas two-headed arrows represent bidirectionality, e.g., improved diagnosis is likely to allow for more fine-tuned structural and functional correlates of DOC and vice versa

In the present white paper, we aim to identify the most pressing current gaps in our understanding of DOC and strategies for closing those gaps. Because DOCs are characterized by heterogeneity, both in phenotype (arousal and cognitive and motor functions) and brain function, understanding the specific neural mechanisms of DOC will require investigation using integrative perspectives. Specifically, we conceptualize three targets for such integration: linking brain structure and function, linking microscale and macroscale levels of analysis, and combining theory-driven and data-driven approaches to scientific discovery. We further envision that enhanced synergy between these three domains will provide a critical vantage point from which to empower diagnosis, prognosis, and treatment of patients, thereby establishing a virtuous cycle between scientific advances and clinical practice (highlighted in Fig. 1).

## Methodology

A 13-member Mechanisms Work Group (co-authors of the present white paper) was convened as part of the Curing Coma Campaign, Coma Science Work Group to identify research gaps and approaches to address these gaps. The work group met weekly from June 19, 2020, to September 4, 2020, to develop consensus recommendations and on an as-needed basis while authoring the current article.

First, literature review and expert discussion were performed by clinical syndrome: coma, vegetative state/unresponsive wakefulness syndrome (VS/UWS), minimally conscious state (MCS), and CMD. After a meeting with the Coma Science Work Group endotype subgroup, a second round of literature review and expert discussion was organized to draft the current recommendations, grouped by research gap theme. The mechanisms are cast in terms of consciousness vs. environmental connectedness vs. responsiveness (see below). To make progress on understanding the relevant mechanisms, we have identified three key gaps that need to be filled (Fig. 1): (1) brain structure vs. brain function, (2) micro- vs macroscale neural mechanisms, and (3) theories vs. data-driven approaches.

## Results of Gap Analysis

### Mechanisms of Interest and Current Gaps: Consciousness, Environmental Connectedness, and Responsiveness

Disorders of consciousness are characterized by a wide array of symptoms and etiologies. Consequently, a number of classification schemes across different dimensions have been proposed that are based on aspects such as cognitive functions [10], awareness [11] and sensory, motor, and arousal behavioral functions [12]. With the acknowledgment that any one framework cannot fully capture all the constructs relevant to DOC, we propose to contextualize our recommendations within the framework provided by Sanders et al. [13], which distinguishes between consciousness, environmental connectedness, and responsiveness (C-EC-R), as outlined below.

Lack of responsiveness remains the clinical criterion for DOC. However, there is a clear conceptual distinction between responsiveness (specifically, nonreflexive behavioral responses) and consciousness (presence of subjective experience, regardless of what the experience is about [13]; note that here we will use this term to also encompass awareness). The possibility for a dissociation between these two dimensions is sharply illustrated by the (rare) case of patients experiencing intraoperative awareness during anesthesia, whose unresponsiveness due to paralysis is mistaken for evidence of unconsciousness [14–16]. Likewise, independent studies during sleep and anesthesia (in which retrospective reports can be obtained on awakening) demonstrate that subjective experience frequently occurs even in the absence of any behavioral responsiveness [17, 18]. Within DOC, routine assessment based on overt responsiveness may be missing covert consciousness in about ~15–20% of patients [3, 4], a fact that has practical and ethical implications for the management of these patients.

**Fig. 2:**
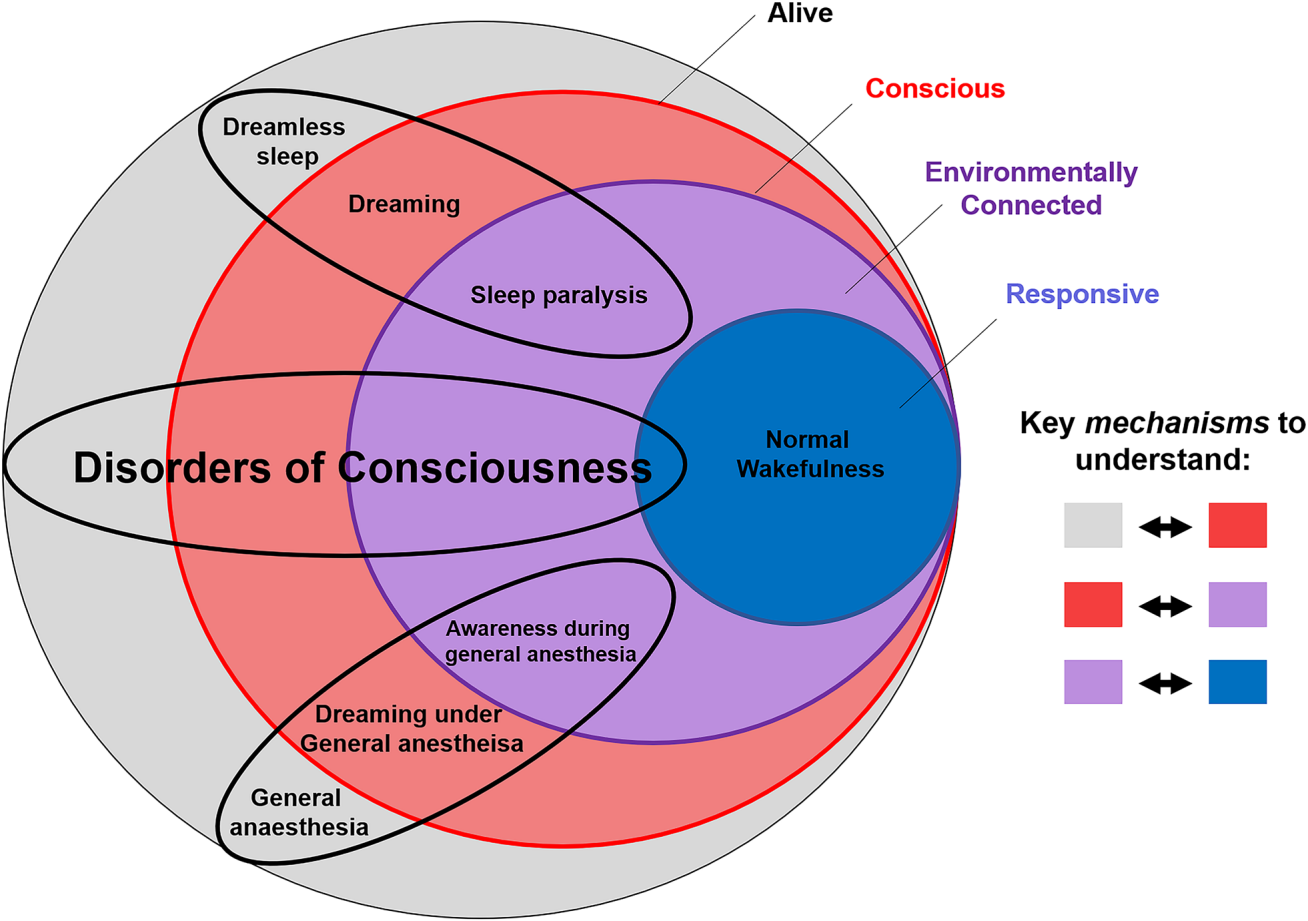
Putative relationships between consciousness, environmental connectedness, and responsiveness (C-EC-R). Illustrative examples are shown pertaining to sleep (top ellipse), general anesthesia (bottom ellipse), and disorders of consciousness (middle ellipse). Note that this is not an exhaustive mapping of all possible states of altered consciousness; likewise, this framework does not directly address the question of quantifying residual cognitive function, as this can only be properly assessed in responsive patients. Also note that the relative size of the colored circles is not intended to reflect relative prevalence

In addition to consciousness and responsiveness, a third relevant dimension is environmental connectedness (as originally proposed in the context of anesthesia [13]). Environmental connectedness corresponds to consciousness of the external environment so that what happens in the environment influences the contents of one’s consciousness (unlike, e.g., during some dream states). On the basis of this framework of C-EC-R, patients with CMD can be characterized as being conscious and environmentally connected but not (overtly) responsive. An intriguingly similar example of dissociation between consciousness and responsiveness is found in patients studied under anesthesia by using the isolated forearm technique, whereby an inflated cuff on the arm prevents it from being paralyzed during general anesthesia [13]. Despite the absence of spontaneous behaviors, such patients can perform simple commands, such as squeezing the physician’s hand (although the potential confound of arousing nociception due to the inflated cuff should also be considered). Examples of conscious but environmentally disconnected states include dreaming and dissociative states induced by ketamine; examples of states that are conscious and environmentally connected but unresponsive are awareness during general anesthesia and sleep paralysis, whereby the paralysis that naturally occurs during rapid eye movement (REM) sleep persists for a short time after awakening [19]. Of course, it is also important to consider the notion of behavioral arousal; although likely neither sufficient for consciousness (given the presence of sleep–wake cycles in patients with VS/UWS) nor necessary for it (given the possibility of dreams during deep non-REM sleep), arousal may nevertheless be a background prerequisite for responsiveness and possibly also for full-fledged environmental connectedness (Fig. 2).

### Current Gaps in Coma Science

Correctly characterizing each patient in terms of the three dimensions described in the C-EC-R framework may be valuable for improving diagnosis and determining the prognosis and appropriate standards of care; for instance, it is especially pressing to establish communication with unresponsive but connected patients and not only identify reliable biomarkers for conscious contents, such as emotional distress or pain (i.e., the conscious processing of nociceptive information), but also identify whether some environmental stimuli trigger them (environmental connectedness). Additionally, by capitalizing on the availability of subjective reports after awakening from sleep or anesthesia in the laboratory environment, it will be crucial to identify ways to detect environmental connectedness based on brain function alone.

By identifying the neural mechanisms that determine transitions between the dimensions of the C-EC-R framework, it may be feasible to devise targeted treatment strategies aimed at restoring each in their own right and to identify which personalized avenues may be the most advantageous for a given patient. Specifically, efforts should be made not only to investigate whether specific mechanisms identified from studies of anesthesia and sleep [13] could also apply to patients with DOC (e.g., to what extent could the isolated forearm scenario constitute a good model for CMD?) but also to investigate how these different states differ from one another (anesthesia is reversible on a short time scale, but DOC may not always be). Additionally, investigations should seek to identify possible dissociations between each of these aspects in subgroups of patients with DOC.

Crucially, the limitations of our present ability to discriminate between elements of the C-EC-R framework in clinical practice are symptomatic of deeper gaps that need to be addressed to obtain a mechanistic understanding of DOC. DOC can arise from a variety of causes, highlighting the need for a more comprehensive understanding of the intricate interactions between structure and function and of their temporary vs. permanent nature. How brain function in turn determines C-EC-R also needs to be clarified, with clear diagnostic utility and prognostic value for recovery. Likewise, a complete understanding of the mechanisms underlying the presence vs. absence of C-EC-R will need to span multiple levels of analysis [20] and biological detail: from the cellular and molecular microscale to macroscale systems and networks. Reconciling the distinct levels of analysis will require the concerted interaction of data-driven approaches combining multimodal data from large-scale studies with theoretical approaches able to integrate their findings into a coherent framework, as well as synthesis through computational modeling. Thus, against the backdrop of the clinical need to discriminate between consciousness, connectedness, and responsiveness in patients with DOC, we envision these complementary approaches as establishing a virtuous cycle to drive forward both scientific understanding and clinical practice.

**Table:** 

**Fig. 3:**
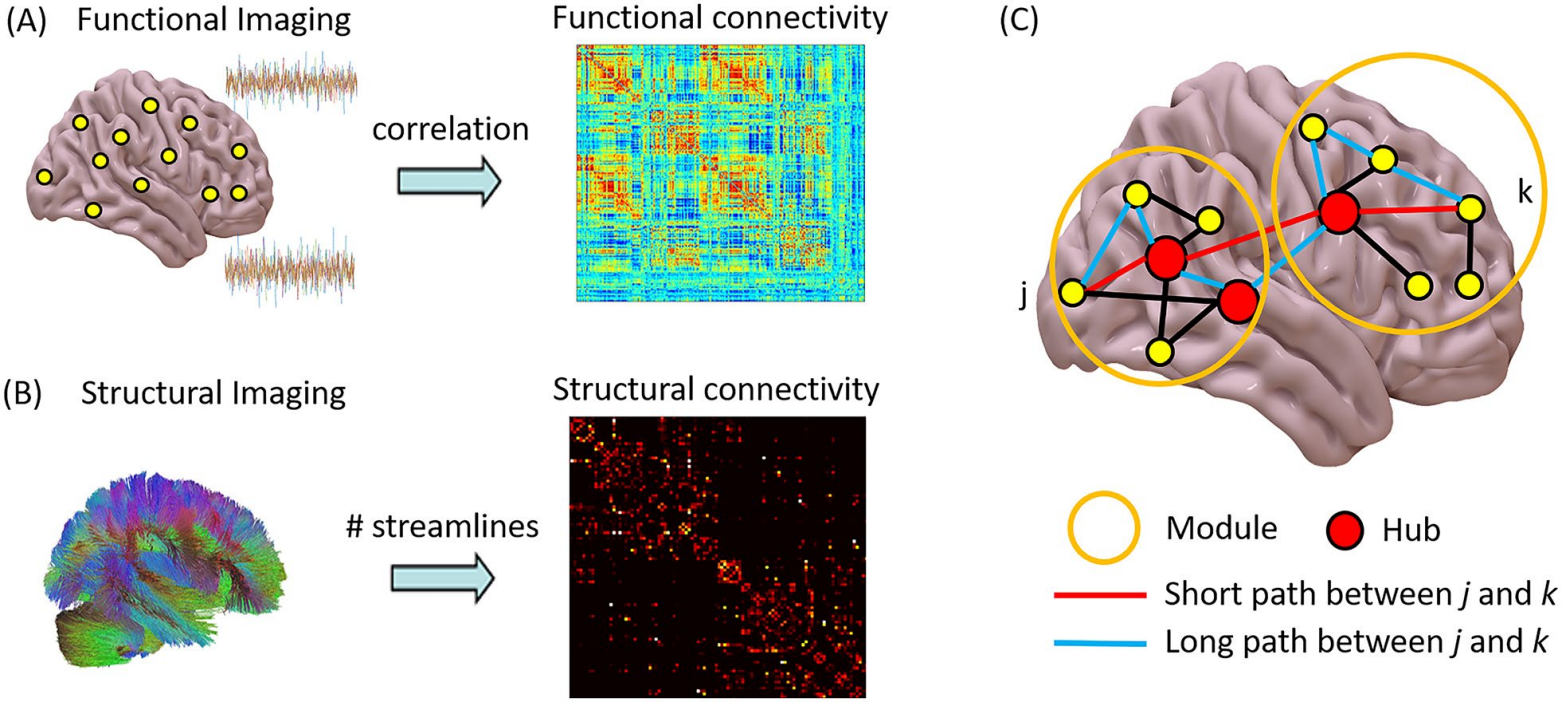
Connectivity in the human brain. **a** Functional connectivity can be quantified from functional neuroimaging, for example, as the Pearson correlation between regional blood-oxygen-level-dependent time series from functional magnetic resonance imaging (MRI). **b** Structural connectivity can be quantified from structural imaging, for example, as the number of streamlines between regions, estimated by using diffusion MRI. **c** Network analysis can provide information about individual nodes (e.g., identification of high-degree nodes, or “hubs”) as well as mesoscopic properties (e.g., modular organization) and macroscale (e.g., average length of shortest path between nodes)

**Box 1** Brain connectivity and networks
The term “functional connectivity” (FC) (Fig. 3a) reflects the notion that similarity between patterns of activity of different brain regions (in terms of statistical dependency) may arise from interactions between those regions. At the spatial and temporal resolutions afforded by noninvasive neuroimaging techniques in humans, neural activity is most frequently estimated in terms of electrophysiology (using EEG or magnetoencephalography) or from the blood-oxygen-level-dependent signal measured by fMRI. Activity is simultaneously measured for each sensor (for EEG/magnetoencephalography) or each voxel (for fMRI) over a period of time.
On the basis of these measurements of brain activity, the most common ways to quantify FC are measures of linear association between pairs of regional time series (primarily, Pearson correlation, but also methods based on phase coherence or spectral properties of the signals), which are therefore agnostic to interactions between more than two elements and ignore the direction of information flow between the two regions. However, more sophisticated measures also exist, capable of addressing various shortcomings of traditional FC (although often at the expense of computational feasibility) [21–26].
Distinct sets of brain regions, termed “resting-state networks,” spontaneously organize into consistently cofluctuating assemblies during both tasks and also at rest. Prominent among these resting-state networks are the frontoparietal control network and the default mode network: these networks typically exhibit inversely correlated time courses at rest [27, 28], but their interactions are consistently perturbed in unconscious individuals [29, 30].
Measures of effective connectivity have also been introduced to identify directed information flow (from region A to region B and not vice versa). Some effective connectivity approaches rely on probabilistic accounts to infer the direction of interactions from statistical relationships in the data (e.g., transfer entropy, Granger causality [31–33]). Another approach to characterize the directionality and strength of interactions, albeit limited to a small number of brain areas, is dynamic causal modeling (DCM), which has also been applied to patients with DOC [34]. The DCM framework is used to infer the direction of connectivity between regions by comparing possible models of how regional signals were generated. First, alternative models are constructed on the basis of possible coupling between regions, viewed as nodes in a directed network. In a second step, the models are compared through Bayesian model selection to identify the model that best explains the empirically observed data [35]. Finally, effective connectivity can be assessed by a perturb-and-measure approach, in which causal interactions are measured by directly stimulating a subset of neurons and by measuring the responses of the rest of the system. In addition to functional and effective connectivity, structural connectivity can also be measured in vivo in humans from diffusion magnetic resonance imaging data (Fig. 3b), for instance, through diffusion tensor imaging (DTI), which can measure the relative diffusion of water molecules along white matter fibers connecting different regions (although without providing information about directionality) [36]. Thus, structural connectivity and FC can be related in the same individual [24, 25, 37, 38].
Whether functional or structural, the interactions between brain regions can be conceived as a network (Fig. 3c), and the mathematical study of networks, known as graph theory, can be used to obtain insights about such networks at multiple levels of resolution [39, 40]: from properties of individual nodes (e.g., degree, measuring how well connected they are [41, 42]) to network modules [26] to macroscale properties such as small-world organization [24, 25, 30, 43–45].

### Brain Structure vs. Brain Function

Advancing the science of DOC requires a precise mapping of the heterogenous structural and functional brain alterations observed in DOC to clinically relevant dimensions (e.g., C-EC-R) across a variety of temporal and spatial scales. Although it is sometimes possible to precisely map neurological dysfunction to a specific location of damaged tissue, it is important to emphasize that brain regions are intricately interconnected, such that local structural or functional changes may well have far-reaching or even global repercussions on other components of the network (diaschisis [46]). Classical lesion-based methods have found success focusing on specific regions of interest (ROI); however, future work should attempt an integration of these approaches with the notion that there exists a many-to-many mapping between brain structure and functional brain states [47]. A full mapping between structural and functional correlates of DOC will require the leveraging of multimodal neuroimaging and neurophysiological techniques, combined with novel analytical methods for integrating them, including the emerging approach of whole-brain computational modeling (Box 2).

Historically, circumscribed lesions and changes in activity within damaged brains have been used to identify ROI and model simplified circuits that may be relevant to DOC symptoms. For instance, the influential mesocircuit model proposes that because of pathological changes following severe brain injuries, a reduction of thalamocortical and thalamostriatal outflow withdraws drive to the frontal cortex and striatum, thus implicating basal ganglia–thalamocortical circuits in the symptoms of DOC [10]. Compared to network or computational perspectives, these focal lesion-based models have the benefit of providing clear targets for treatment (e.g., via deep brain stimulation, low-intensity-focused ultrasound, pharmacological). For instance, emphasis on the role of the thalamus in DOC has compelled the development of techniques for its stimulation, which have been associated with improved behavioral responsiveness in a subset of patients with DOC [9, 48]. Yet the precise functional roles of key ROI, such as the thalamus, remain far from fully characterized; indeed, it remains unclear why nearly full ablation of the thalamus does not appear to result in a loss of consciousness in rodent models [49] despite its consistent association with DOC. Thus, focal characterization of structure–function relationships retains unique value but must continue to be refined.

For instance, although the original mesocircuit model emphasizes pallido-thalamo-cortical communication, this model has been updated by the recent discovery of direct structural connections between the globus pallidus and the cortex, first in rodents [50] and then in humans [51] by using DTI. In parallel, DCM (see Box 1) was applied to the mesocircuit and specifically implicated pallidocortical communication in the transition between states of consciousness under anesthesia [52]. This example illustrates the kind of multidisciplinary workflow that should inform the placement of ROI within increasingly complex frameworks (network, computational) for understanding the structural and functional relationships underlying DOC.

Compared to ROI approaches, the structural correlates of DOC may be further detailed by employing mass-univariate, voxel-wise analysis, which can produce mappings of behavioral symptoms to structural alterations at the millimeter scale (e.g., voxel-level symptom mapping [53, 54]; also see voxel-level shape analysis [55, 56]). Similar approaches have been applied to the structural connectome derived from DTI (known as connectome-based lesion symptoms mapping [57, 58]). Although these methods allow for a more spatially precise connection between large-scale functions (e.g., behavioral arousal) and structural damage, they do not capture the perhaps more elusive reorganizations in functional networks that often follow structural insult and that ultimately produce changes in behavior (e.g., diaschisis) as well as inadequate or maladaptive compensatory mechanisms, which may also produce DOC symptoms. Indeed, regions that are only secondarily affected may be particularly promising treatment targets because of their relative retained structural integrity, which may hold greater potential to reach preinjury levels of functioning. To capture the relationship between gray matter atrophy, white matter disconnection, and functional interactions, there is a need to better integrate structural correlates with the full range of functional modalities available to us instead of behavioral symptoms alone.

A jumping-off point for multimodal integration in DOC may be to overlay the structural correlates of DOC with the healthy human connectome (both structural and functional) to derive likely locations for a diaschisis effect, a method that avoids the often challenging process of multimodal data collection within patients themselves [59, 60]. Such an approach could be used to build whole-brain computational models, including the known structural correlates of DOC and known large-scale functional correlates (Box 2).

**Table:** 

**Fig. 4:**
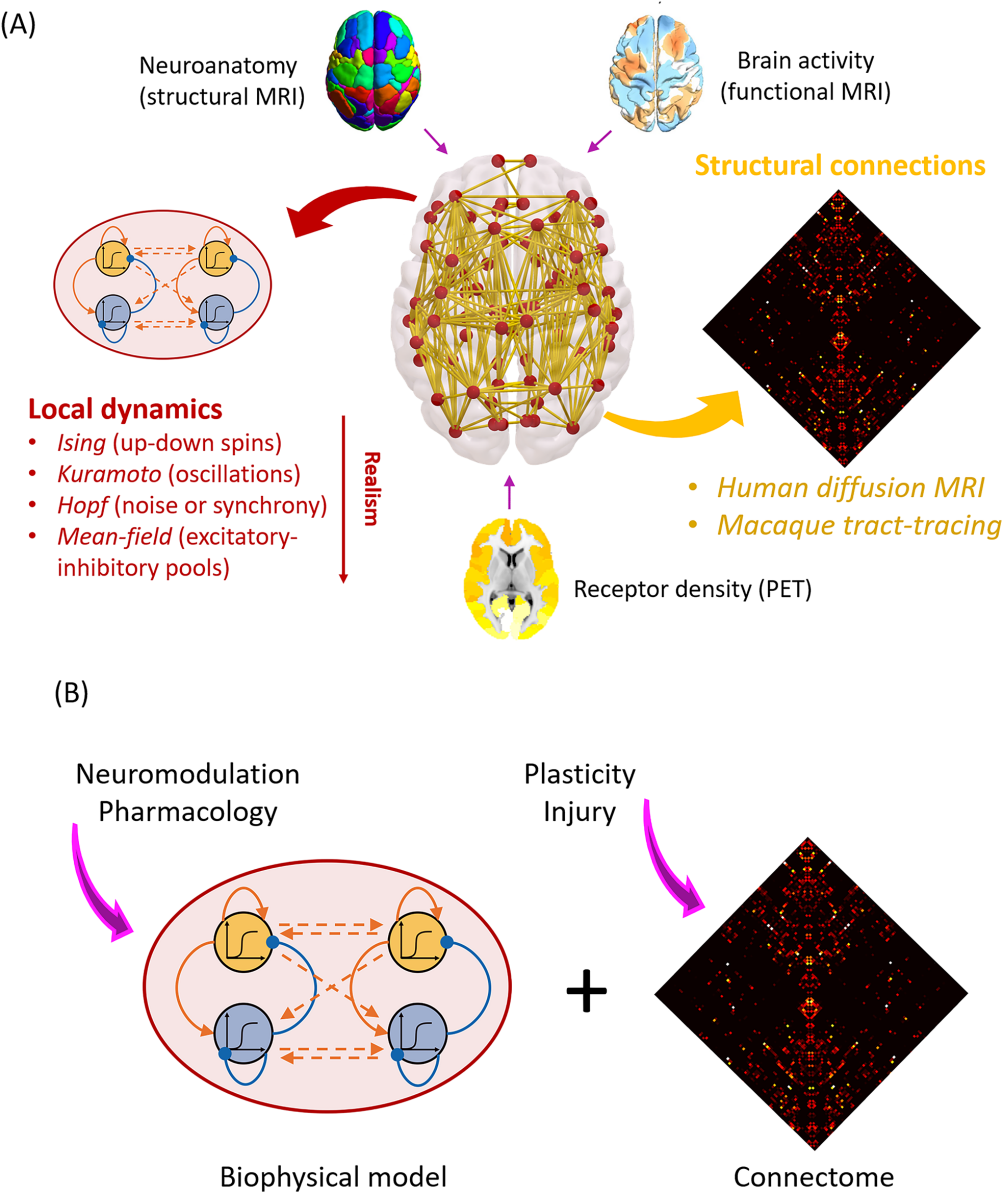
Overview of whole-brain computational modeling to integrate multimodal and multiscale data. **a** Whole-brain models combine a model of local regional activity with information about connectivity between regions. Additional information can be provided about neuroanatomy (from structural magnetic resonance imaging [MRI]), brain function (from functional MRI), and neurobiology (e.g., receptor density distribution obtained from in vivo positron-emission tomography [PET]). **b** Models can be systematically perturbed at different spatial and temporal scales, intervening at the level of individual regions or their connections

**Box 2** Whole-brain computational models
Whole-brain computational models represent a powerful set of tools to study macroscale mechanistic questions in neuroscience [62–64]. Such models typically combine two fundamental ingredients (Fig. 4a): (1) information about brain network structure (e.g., obtained from diffusion-weighted imaging in humans or invasive tract tracing in animals) and (2) a model of regional neural activity, ranging from Kuramoto or Hopf oscillators to the dynamic mean-field model obtained by mean-field reduction of integrate-and-fire spiking neurons with excitatory and inhibitory populations [65]. The complex interactions of these two key components can give rise to rich and biologically realistic functional dynamics analogous to those observed from fMRI and EEG [65, 66]. Although the required level of neurobiological detail will vary according to the specific question under investigation, the more biologically inspired models (e.g., dynamic mean-field) can also be enriched with further information, such as regional myelination or the regional distribution of specific receptors obtained from positron-emission tomography (PET) [64, 67–70]
Importantly, in silico computational models offer several advantages: their parameters are fully available to inspection and manipulation by the researcher, and they can be perturbed in ways that would not be possible in either humans or animals [67–70]. Computational modeling allows formulation and testing of specific mechanisms, a key feature not provided by other techniques (e.g., neuroimaging). Moreover, the same model can be subjected to different kinds of perturbations to investigate which pharmacological or structural interventions have equivalent results on the model’s function (Fig. 4b), a powerful avenue to interrogate potential similarities between anesthesia and DOC. Finally, the advent of computational models offers the unique promise to develop personalized models from each patient’s multimodal neuroimaging data, and subsequently perform systematic perturbation of the model to evaluate the potential effects of different treatment approaches, with the ultimate goal of informing which therapeutic modalities may be applicable for a patient

However, multimodal data collection in patients with DOC—and new methods for merging modalities with minimal information loss [60]—will be necessary to account for the inevitable translational gaps between healthy patients, computational models, and real patients with DOC. Some recent studies have pioneered these approaches; for instance, EEG markers have been linked to subcortical damage in acute [71] and chronic [72] DOC, and recent evidence indicates that preserved fractal (self-similar) character of structural brain networks is associated with covert consciousness on the basis of fMRI response to mental imagery tasks [73]. Inspiration for future joint investigations of structure and function may be drawn from other models of disrupted consciousness, such as anesthesia, in which links between structural and functional networks have recently been identified [24, 38].

To achieve clinical relevance, it will be crucial not only to collect increasingly abundant and multimodal data but also to distill from them the biomarkers that are most predictive of consciousness, connectedness, and responsiveness, either in terms of brain states or information structures, which may better explain *how* functions such as C-EC-R emerge instead of only from *where* [62]. For instance, the framework of connectome harmonic decomposition [47, 74] allows for functional brain activity to be decomposed in terms of different contributions of the human structural connectome [47]. Such connectome harmonics are wave-like patterns of spatial oscillations that represent how information spreads over the structural connectivity of the human brain. Through this approach, a common neural signature was recently identified between reduced consciousness in DOC and under anesthesia [74] and thus may represent a general signature of consciousness that can inform theoretical interpretations.

Of course, a plethora of other measures have emerged in recent years to describe network properties, including graph-theoretic and information-theory-based measures. The perturbational complexity index (PCI), which measures the complexity of EEG signals following causal perturbation by pulses of transcranial magnetic stimulation (TMS), appears particularly adept at detecting covert consciousness during sleep and anesthesia, as well as in patients with DOC, without the need for behavioral response [6]. Other measures of this kind, although perhaps less accurate for diagnosis, have also shown significant prognostic value [41, 75]. Future approaches should seek relationships and convergence between metrics derived from information theory, graph theory, and dynamical systems theory and strive to connect them with structural measures. Contemporary examples include the recent link found between PCI and subcortical atrophy [76] and the recent association between focal cortical lesions and the generation of pathological slow waves, disconnection, and lost complexity [77, 78].

Alongside sleep or anesthesia, seizures present yet another way to interrogate the generalizability of brain states to the C-EC-R framework in unique contexts (e.g., presence of fast-spiking high metabolism in seizures compared with anesthesia and DOC). This is especially relevant given that specific deficits in C-EC-R can all present during seizures in patients with chronic epilepsy [79–81]. The loss of informational complexity typically observed during seizures [82] and the restored behavioral arousal following subcortical (pons, thalamus) stimulation during focal seizures in rats [83] suggests that generalizability is likely to be found; however, this area is ripe for more investigation.

Importantly, the first steps toward the integration of structure and function in DOC are largely taking place by experimental, analytic, and computational integration of the various neuroimaging methods that probe macroscale phenomena. However, a full mapping of structure–function relationships must inevitably dive deeper into the microscale neural substrate on which macroscale networks arise. Indeed, modeling the biological correlates of DOC across all spatial scales is likely to improve the location of individual patients within the heterogeneous space of DOC manifestations (e.g., as defined by C-EC-R). Thus, in the next section, we detail the gaps that exist in our understanding of microscale neural underpinnings of DOC and coma and how macro and micro levels of understanding may be bridged.

## Linking Micro- and Macroscales

A comprehensive understanding of DOC requires that insights from macroscopic and mesoscopic levels of inquiry sampled by neuroimaging are integrated with insights from more microscopic scales: the relevant contributions at the systems, cellular, genetic, and molecular levels. Because these can largely only be studied directly in animal models, bridging this gap necessitates a closer association of preclinical and clinical research and the generation of hypotheses that can be bidirectionally tested in both animal and human research approaches.

Animal models that allow relevant direct measurements of such microscopic facets remain limited for coma [84] and fully absent for VS/UWS and MCS. The development of functionally relevant animal models will need to distinguish between the various neurological events (e.g., traumatic brain injury, anoxia, hypoxia among others) associated with DOC and the subsequent neurological syndromes of DOC (e.g., MCS, UWS/VS) [85, 86] to characterize both etiology-specific and generalizable DOC mechanisms. This could be further enhanced through comparison with results from sleep and anesthesia research, in which microscopic mechanisms have been more comprehensively probed in preclinical work [87], producing mechanistic frameworks that span from whole-brain phenomena, such as individual susceptibility, to anesthetic-state transitions (neural inertia), all the way to genetic susceptibility factors for anesthesia [38, 88, 89]. Indeed, work in rodents and cortical slices alike has recently demonstrated that neuronal “off” periods determine a dramatic collapse of large-scale interactions and complexity during non-REM sleep and anesthesia [90–92], which can also be assessed by using EEG coupled with TMS in humans with brain damage [77, 78].

In terms of experimental approaches, novel noninvasive in vivo optogenetic techniques allow for the modulation of very specific and deeply seated targets in animal models [93, 94]. Leveraging these technological developments will enable systematic hypothesis-driven investigations of mechanisms that have been suggested both in previous anesthetic, sleep, and lesion studies in both animals and humans. Importantly, combining optogenetic stimulation with simultaneous high-resolution neuroimaging [95] could provide biomarkers to serve as direct translational interfaces between animal and human research.

Firstly, a systems-level microscopic perspective requires a comprehensive investigation of subcortical structures in neuroimaging given the implications of these structures in animal and translational research across trauma, anesthesia, and sleep. Specifically, the historical dichotomy between the cortex as the substrate of contents of consciousness and the thalamus and brainstem as substrates of arousal has oversimplified the various and diverse roles of subcortical systems [96]. As a first step, efforts should be directed toward a detailed mapping of how key subcortical structures (e.g., thalamus, brainstem nuclei, and basal ganglia) interact with specific cortical layers by using high-field structural and functional neuroimaging and complementary animal models [97, 98]. A relevant example of how current oversimplified views of the subcortical–cortical interplay can be refined into region- and lamina-specific accounts is provided by Redinbaugh et al. [97], who used thalamic stimulation in the anesthetized macaque to reveal that consciousness-relevant thalamic influence differs between deep and superficial cortical layers.

Similarly, critical insights at the microscopic systems level are to be gained from the brainstem neuromodulatory nuclei, which have been extensively studied in animals by using anesthetic and lesion approaches [99–101]. Their associated transmitter systems and brain-wide neuromodulatory projections have been variously implicated as causing coma [60, 102, 103]. In healthy patients, they have been found to possibly drive both tonic and phasic large-scale in vivo brain activity [104–106]. Established and novel single-photon emission computed tomography approaches [107] and innovative magnetic resonance sequences (e.g., magnetization transfer images [108]), combined with increased magnetic resonance field strengths, will allow these nuclei and their brain-wide projections to be more directly probed. These approaches should delineate whether dysfunction of these nuclei alters their modulation of the whole-brain connectome, which in turn may cause the striking macroscopic network disruptions commonly observed in DOC. This should use complementary approaches in both animal and patient research as well as computational modeling. Specifically, recent identification of the dopaminergic system’s relevance for wakefulness from anesthesia models has begun to provide a mechanistic foundation of why dopamine may have emerged as a key pharmacological treatment target in DOC [99, 109–112]. To maximize therapeutic potential, comprehensive assessments of associated brainstem nuclei should also aim to delineate whether their dysfunctions in patients with DOC are pre- or postsynaptic using approaches, such as those demonstrated by Fridman et al. [113], combining PET with bolus administrations of pre- or postsynaptically acting pharmacological agents to characterize a presynaptic dopaminergic release deficit in patients with DOC.

Further progress along the microscopic perspective requires that differential involvements of particular neuronal types and subtypes in consciousness alterations be delineated. These studies will largely have to be delivered by using single-unit recordings and conditional expression approaches in novel animal models to delineate the relevance of neuronal types, e.g., pyramidal neurons [114]. For instance, anesthetic-induced decoupling between apical and basal compartments of layer 5 pyramidal neurons in rodents impairs large-scale functional integration in the brain [115]. These animal experiments can, however, also be complemented by work in human neuroimaging by using whole-brain transcriptomic maps from microarray data, as demonstrated by Craig et al. [116], who used this approach to delineate that GABAergic cortical interneuron subtypes are differently affected in anesthesia.

Indeed, it is key to not only characterize the involvement of different neuronal subtypes but equally characterize the role of glia and glial subtypes in DOC [117], especially given that widespread white matter damage is commonly associated with these conditions [118, 119]. Both animal models of glial modulation and alteration in response to trauma/anoxia [120] and new approaches in future neuroimaging studies [121] will have to constructively integrate white matter structure and function [121, 122]. An area of particular promise is the study of how microglia mediate systemic and regional inflammation following both traumatic brain injury and anoxia [123], leading to DOC. PET ligands for activated microglia and concomitant longitudinal collection of inflammatory and cell death markers in blood and/or cerebrospinal fluid should also distinguish inflammation in acute and chronic phases of DOC [124]. Animal models should aim to distinguish neuroprotective and pathogenic inflammatory effects in vivo [125] and distinguish whether pharmacological intervention can induce neuroprotective states, leading to more favorable outcomes. Similar multimodal techniques can also be used to begin to probe the roles of other glial cell types, such as astrocytes, whose role in overall homeostasis of the central nervous system has been demonstrated in traumatic brain injury and thus may play a similar role on the DOC spectrum [126, 127].

**Fig. 5:**
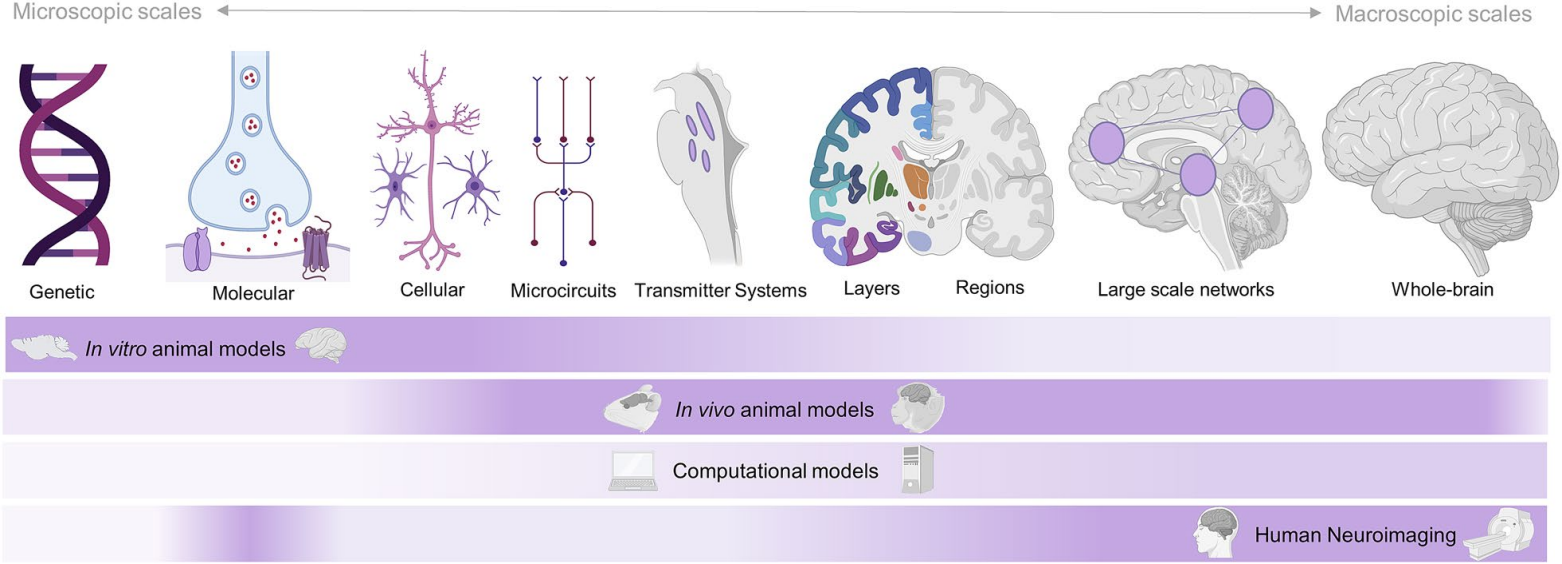
Conceptual overview of levels of analysis to be considered in disorders of consciousness (DOC) research across the microscopic-to-macroscopic spectrum. Gradients indicate the capability of a technique to make measurements relevant to the level indicated above, thus highlighting gaps and possible translational interfaces. Human neuroimaging has produced macroscopic network biomarkers and certain regions/layers whose disruption is associated with DOC. For inquiries at more microscopic scale, animal models are indispensable, in which experimental manipulations (DREADD, optogenetics, lesion approaches, etc.) allow for direct mechanistic investigations, which can produce insights that can in turn be tested in humans in vivo (e.g., by using pharmacological approaches). The wider usage of high-field neuroimaging in both humans and animals will produce particularly relevant integrations of these levels, which will also serve to produce the type of data required to enable the generation of truly mechanistic computational approaches (e.g., whole-brain modeling). Altogether, these levels of analyses and models are complementary and synergistic for the discovery of the biological mechanisms of DOC

Finally, the subcellular and thus most microscopic level holds great promise for future explorations. In particular, it is necessary to identify genetic and molecular mediators of clinical progression and outcome in DOC. Beyond relying on analogies from anesthesia, it is required to delineate specific receptors and particular pathways that may be associated with the development or persistence of DOC. Although no specific genetic factors have yet been associated with DOC, genetic risk factors for adverse outcomes in traumatic brain injury/anoxia serve as useful and possibly intertwined starting points [128]. However, because human genome-wide association studies require prohibitively large population sizes, insights across all other levels of microscopic analysis (see Fig. 5) should be combined into biologically and physiologically relevant collections of gene candidates to be assessed with approaches such as transcriptome-wide association studies [129]. These could identify viable genetic candidates that in turn could be incorporated into both animal (assessing, e.g., loss-of-function and gain-of-function alterations) and human experiments (e.g., using microarray data [116]), thereby feeding back relevant, testable knowledge across all levels of analysis at which we need to consider DOC mechanisms as well as the theoretical frameworks that build on them.

## Theory vs. Data-Driven Approaches

Recent research continues to enable a better understanding of the neuronal causes and clinical manifestations of DOC. Among the approaches that have recently been developed, we may distinguish purely empirical ones and those driven by assumptions of certain theories of consciousness. However, in their current state, it seems that both theories and data-driven approaches are unable to make predictions precise enough to enable a clear distinction between the neural mechanisms of the three dimensions of the C-EC-R framework. Yet this mechanistic understanding would carry immense clinical and ethical significance. For example, one of the key issues for clinicians is the ability to better predict the presence vs. absence of pain in unresponsive patients [130] and what may trigger it. Obtaining such diagnostic biomarkers would require a mechanistic understanding of the foundations of presence vs. absence of both consciousness and environmental connectedness [13] without reliance on behavior alone. Thus, a convergence between data-driven metrics (behavioral and neural) and theoretical perspectives on consciousness and environmental connectedness is needed to improve our ability to estimate the degree of residual consciousness and environmental connectedness (e.g., the presence of suffering) in unresponsive patients.

Recently, we have witnessed a formidable growth of new metrics based on neuroimaging [131] for the diagnosis of consciousness and the prognosis of recovery after severe brain injury. For example, various studies have shown a reduction in entropy (a measure of signal diversity or unpredictability) when consciousness fades, e.g., in sleep, anesthesia, and DOC [30, 61, 132, 133]. Although measures of this kind may demonstrate impressive predictive power, it remains unclear if certain metrics are relevant for consciousness per se or rather only represent epiphenomenal correlates (e.g., decline in entropy might be observed when transitioning from fast to slow oscillatory activity during anesthesia), and the above-mentioned EEG complexity has also been dissociated from responsiveness in anesthetized rodents [134]. Thus, although appealing, relying on promising but theoretically ambiguous data-driven metrics in a clinical setting runs the risk of introducing a discrepancy between what is intended to be detected (e.g., consciousness) and what is being measured; a similar problem is the so-called black box in medical artificial intelligence [135], whereby classification (diagnosis) and prediction (prognosis) can be successful but for opaque reasons [135].

Various prominent theories of consciousness tend to emphasize different neurophysiological underpinnings. For example, integrated information theory (IIT) suggests that consciousness requires a specific kind of causal interaction between elements of the system capable of supporting the integration of information [136]. On the basis of its early theoretical concepts [137–139] that explicitly linked consciousness to complexity, defined as coexistence of functional differentiation and functional integration in the brain, measurement of EEG responses to magnetic perturbation (TMS) led to successful detection of residual awareness after brain injury (PCI [5]). Alternatively, the global neuronal workspace theory proposes that information processed in parallel by specialized modules needs to compete for access to a global workspace of frontoparietal circuits, whereupon it is integrated and subsequently broadcasted to the entire brain, becoming available for conscious processing [140–142]. Some measures derived from graph theory (e.g., “small-worldness,” which reflects the balance of local segregation and global integration in a network [40]) have been argued to capture a decrease in such broadcasting in conditions of fading responsiveness [30, 43, 45, 143, 144]. However, there is still a need to more specifically associate these theoretical frameworks (among the many others) with concrete biomarkers so that empirical data may more clearly inform on the validity of particular theoretical interpretations over others. To do so, there is a pressing need to explicitly clarify, for example, the constructs of integration and information, as featured in different theoretical accounts, as well as their underlying assumptions.

In response to the current gaps and inconsistencies identified above, both data- and theory-driven approaches should advance toward unified terminology and contents that can be readily compared. First, we should aim for precise theoretical predictions for environmental disconnection and unconsciousness that are distinct from behavioral responsiveness, both at the information level and in terms of possible biological mechanisms. Indeed, there can be distinct biological mechanisms leading to the same functional or informational end point (e.g., multiple possible causes leading to brain states of low complexity). Both data- and theory-driven biomarkers for DOC should first be verified in conditions in which subjective reports are available (sleep, anesthesia, seizures) and then be precisely described in relation to their target: the distinction in C-EC-R (see Fig. 2).

To ameliorate the inconsistencies outlined here, theories should help to identify markers that may be most discriminative, and in the case of existing data-driven markers, their proponents should be able to explain their relevance to particular theoretical perspectives. Ideally, data-driven approaches should focus on a comprehensive set of markers indexing different biological levels, e.g., from metabolism and resting-state EEG/fMRI measures to effective connectivity or task-based paradigms. Similarly, comprehensive theoretical frameworks should span different descriptive levels (e.g., circuit level, information level, and topological level) and explicitly connect their predictions to different types of empirical markers.

We acknowledge that comparisons between existing theories are challenging because they sometimes operate on different definitions of consciousness. Furthermore, biological mechanistic frameworks, such as the mesocircuit hypothesis [10], may be compatible with a mathematical framework, such as IIT, when viewed as providing the required background conditions (e.g., neuromodulators maintaining adequate excitability) that enable the physical substrate of consciousness itself to function [76, 145]. Although some recent efforts have attempted to reconcile concepts derived from different theories of consciousness [146, 152], a direct involvement of the proponents of each theory in an adversarial context is most helpful to identify commonalities and differences in theoretical predictions and come up with specific experiments through which their predictions can be explicitly compared. For example, some incompatible predictions of global neuronal workspace and IIT are now being explicitly tested [147]. Ultimately, such adversarial collaboration may provide different degrees of data-driven support for different theoretical frameworks to make inferences about the presence of consciousness and/or environmental connectedness in patients with DOC.

## Conclusion and Future Directions

In this position paper, we suggest that bringing together and integrating different levels of analyses and modalities, different scales, and different approaches, while at the same time attending to discrete relevant dimensions (C-EC-R), will prove a challenging but vital approach to push forward our mechanistic understanding of DOC (Table 1).

**Table 1:** Future research needs for investigating mechanisms of consciousness toward improved diagnosis, prognosis, and treatment of DOC

Research need: establish a framework for differentiating clinical subtypes of DOC with concepts of C-EC-R
**Research need: identify links between structural brain damage and abnormal brain function in DOC**
Form a complete mapping of DOC structural damage to functions of interest across multiple temporal and spatial scales
Identify structural correlates of relevant biomarkers of brain function in DOC patients derived from different approaches, e.g., information theory, graph theory, and dynamical systems theory
**Research need: provide an integrated understanding of DOC across biological micro- and macroscales**
Develop clinically relevant animal models of DOC for translational research approaches
Identify the role of subcortical structures and their interplay with the cortex in heterogeneous DOC
Associate microscale (neuronal and nonneuronal), and subcellular (molecular and genetic) mediators, with in vivo manifestations in patients with DOC
**Research need: induce integration between theory-driven and data-driven approaches**
Develop precise theoretical predictions and further biomarkers to address each dimension of the C-EC-R framework
Build a comprehensive set of data-driven and theory-driven biomarkers addressing different levels of analysis
Compare and develop existing theories in adversarial collaboration between theory leaders
**Research need: integrate levels of description, imaging modalities, and theoretical approaches**
Analyze multilevel and multimodal data from large-scale data sets to construct more realistic computational models of DOC
Develop personalized medicine models to guide treatment based on individual patients’ multimodal data

*C-EC-R* consciousness, environmental connectedness, and responsiveness, *DOC* disorders of consciousness

To achieve these ambitious goals, the field will need to leverage multimodal data in the same patients over time but also in diverse patients and across different states of altered consciousness (e.g., sleep, anesthesia, seizures), as well as in animal models, to enable the generalization of results. Several categories of techniques stand out for their ability to provide both improvements within each area and further integration between them, namely, (1) increasingly multilevel animal models, (2) analytic techniques for aggregating the various neural correlates of DOC (e.g., machine learning), and (3) increasingly multilevel whole-brain computational models.

Although direct animal models of DOC remain absent and our ability to disentangle consciousness from responsiveness in animals remains limited because of subjective reports being unattainable, animal models continue to be an important avenue for expanding our understanding of the biological mechanisms of DOC. Specifically, the expansion of multimodal methods for experimental manipulation in animals provides opportunities to selectively probe mechanisms and neuronal populations in the brain. Such work in animals could provide translational bridges from foundational microscale alterations and effectors to the neuroimaging findings commonly observed in patients with DOC [148] and thus catalyze the identification of translatable treatment targets and strategies.

To make sense of the wealth of data that such increasingly multimodal and multivariate approaches will produce, interrogation through new analytic tools will also be called for. Machine learning based on neuroimaging data has already been used to predict prognosis in patients with DOC [41, 42] as well as to differentiate states of consciousness (wakefulness from DOC or anesthesia) [75]. Nevertheless, predictive models derived from machine learning can help to both reduce redundancy between metrics and identify synergy between many identifiable neural correlates of DOC to distill clinically relevant predictions (e.g., probability of recovery, likelihood of covert consciousness).

In this vein, the increasing richness of empirical data can be leveraged to develop increasingly realistic computational models of DOC. In silico whole-brain models offer an especially promising avenue, with their potential to combine macroscale information about brain structure and function with considerations of microscale neurobiology [62–64] (Box 2) as well as related empirical data and theoretical perspectives at multiple levels (e.g., how systems-level accounts of DOC interact with information perspectives). Indeed, successful models of anesthetic states have already been put forth [149–151]. Therefore, computational models could provide means to address each of the three gaps we have identified, with the potential to aid personalized medicine.

Throughout this article, we have emphasized that closing the gaps we have identified must be understood as a synergistic endeavor. Addressing gaps at one level (e.g., the relationship between micro- and macroscale) will also inform our understanding of the interactions between brain structure and function. At the same time, closer translational interfaces between these areas of study will thereby inevitably emerge. Likewise, in addition to addressing the specific questions and challenges outlined here, large-scale multimodal data sets across humans and animals will provide the basis on which data-driven approaches and subsequent modeling can be developed further and refined. Finally, bidirectional interaction between scientific investigation and clinical practice will continue to play a fundamental role, with models and biomarkers informing prognosis and treatment while being informed by clinical insight, engendering a virtuous cycle. Nevertheless, we do not consider bridging these gaps as the end point of coma science but only as a new vantage point—a vantage point of integrated knowledge across levels, imaging modalities, and theoretical approaches—from which to pursue our goal: curing coma.
