# Correction: Mechanisms Underlying Disorders of Consciousness: Bridging Gaps to Move Toward an Integrated Translational Science

**DOI:** 10.1007/s12028-022-01488-1

**Published:** 2022-04-01

**Authors:** Andrea I. Luppi, Joshua Cain, Lennart R. B. Spindler, Urszula J. Górska, Daniel Toker, Andrew E. Hudson, Emery N. Brown, Michael N. Diringer, Robert D. Stevens, Marcello Massimini, Martin M. Monti, Emmanuel A. Stamatakis, Melanie Boly, the Curing Coma Campaign and Its Contributing Collaborators

**Affiliations:** 1 Division of Anaesthesia, School of Clinical Medicine, University of Cambridge, Cambridge, UK; 2 Department of Clinical Neurosciences, University of Cambridge, Cambridge, UK; 3 Department of Psychology, University of California, Los Angeles, Los Angeles, CA USA; 4 Department of Psychiatry, University of Wisconsin–Madison, Madison, WI USA; 5 Department of Anesthesia and Perioperative Medicine, David Geffen School of Medicine, University of California, Los Angeles, Los Angeles, CA USA; 6 Department of Anesthesia, Critical Care, and Pain Medicine, Massachusetts General Hospital and Harvard Medical School, Harvard University, Boston, MA USA; 7 Department of Brain and Cognitive Sciences, Massachusetts Institute of Technology, Cambridge, MA USA; 8 Department of Neurology and Neurosurgery, Washington University in St. Louis, St. Louis, MO USA; 9 Departments of Anesthesiology and Critical Care Medicine, Neurology and Neurosurgery, and Radiology, School of Medicine, Johns Hopkins University, Baltimore, MD USA; 10 Dipartimento di Scienze Biomediche e Cliniche “L. Sacco”, Università Degli Studi Di Milano, Milan, Italy; 11 Istituto Di Ricovero e Cura a Carattere Scientifico, Fondazione Don Carlo Gnocchi, Milan, Italy; 12 Brain Injury Research Center, Department of Neurosurgery, David Geffen School of Medicine, University of California, Los Angeles, Los Angeles, CA USA

## Correction to: Neurocrit Care (2021) 35:37–54 10.1007/s12028-021-01281-6

The authors would like to correct their conflict of interest declaration. It should read: Dr. Emery N. Brown declares he is a cofounder of PASCALL Systems, which is a startup for a next generation wearable brain monitoring system for patient care. The other authors declare no conflicts of interest.

